# Supplementary material for: Understanding the risks for post-disaster infectious disease outbreaks: a systematic review protocol
Source: BMJ Open. 2020 Sep 13;10(9):e039608. doi: 10.1136/bmjopen-2020-039608 (PMC7488804; doi:10.1136/bmjopen-2020-039608)
Supplement: Author's manuscript [file bmjopen-2020-039608.draft_revisions.pdf]

BMJ Open is committed to open peer review. As part of this commitment we make the peer review history of every article we publish publicly available.

When an article is published we post the peer reviewers' comments and the authors' responses online. We also post the versions of the paper that were used during peer review. These are the versions that the peer review comments apply to.

The versions of the paper that follow are the versions that were submitted during the peer review process. They are not the versions of record or the final published versions. They should not be cited or distributed as the published version of this manuscript.

BMJ Open is an open access journal and the full, final, typeset and author-corrected version of record of the manuscript is available on our site with no access controls, subscription charges or pay-per-view fees (http://bmjopen.bmj.com).

If you have any questions on BMJ Open's open peer review process please email info.bmjopen@bmj.com

Understanding the risks for post-disaster infectious disease outbreaks: a systematic review protocol

Journal:	*BMJ Open*
Manuscript ID	bmjopen-2020-039608
Article Type:	Protocol
Date Submitted by the Author:	20-Apr-2020
Complete List of Authors:	Charnley, Gina; Imperial College London Faculty of Medicine, School of Public Health, Department of Infectious Disease Epidemiology<br>Gaythorpe, Katy; Imperial College London Faculty of Medicine, School of Public Health, Department of Infectious Disease Epidemiology<br>Murray, Kris; Imperial College London Faculty of Medicine, School of Public Health, Department of Infectious Disease Epidemiology<br>Kelman, Ilan; University College London Faculty of Mathematical and Physical Sciences, UCL Institute for Risk and Disaster Reduction; University College London Faculty of Population Health Sciences, UCL Institute for Global Health
Keywords:	Protocols & guidelines < HEALTH SERVICES ADMINISTRATION & MANAGEMENT, Epidemiology < INFECTIOUS DISEASES, PUBLIC HEALTH, Epidemiology < TROPICAL MEDICINE

SCHOLARONE™
Manuscripts

I, the Submitting Author has the right to grant and does grant on behalf of all authors of the Work (as defined in the below author licence), an exclusive licence and/or a non-exclusive licence for contributions from authors who are: i) UK Crown employees; ii) where BMJ has agreed a CC-BY licence shall apply, and/or iii) in accordance with the terms applicable for US Federal Government officers or employees acting as part of their official duties; on a worldwide, perpetual, irrevocable, royalty-free basis to BMJ Publishing Group Ltd ("BMJ") its licensees and where the relevant Journal is co-owned by BMJ to the co-owners of the Journal, to publish the Work in this journal and any other BMJ products and to exploit all rights, as set out in our licence.

The Submitting Author accepts and understands that any supply made under these terms is made by BMJ to the Submitting Author unless you are acting as an employee on behalf of your employer or a postgraduate student of an affiliated institution which is paying any applicable article publishing charge ("APC") for Open Access articles. Where the Submitting Author wishes to make the Work available on an Open Access basis (and intends to pay the relevant APC), the terms of reuse of such Open Access shall be governed by a Creative Commons licence – details of these licences and which Creative Commons licence will apply to this Work are set out in our licence referred to above.

Other than as permitted in any relevant BMJ Author's Self Archiving Policies, I confirm this Work has not been accepted for publication elsewhere, is not being considered for publication elsewhere and does not duplicate material already published. I confirm all authors consent to publication of this Work and authorise the granting of this licence.

For peer review only

Understanding the risks for post-disaster infectious disease outbreaks: a systematic review protocol

Gina E. C. Charnley[1], Katy Gaythorpe[1], Kris A. Murray[1], Ilan Kelman[234]
[1]Faculty of Medicine, School of Public Health, Imperial College London, London, UK. [2]Faculty of Mathematical and Physical Sciences, UCL Institute for Risk and Disaster Reduction & [3]Faculty of Population Health Sciences, UCL Institute for Global Health, University College London, London, UK. [4]University of Agder, Kristiansand, Norway.
Correspondence to Gina Charnley; g.charnley19@imperial.ac.uk; School of Public Health, St. Mary's Campus, Imperial College London, London, W2 1NY.
Word Count 2,133

ABSTRACT
Introduction
Disasters have many forms, including those related to natural hazards and armed conflict. Human-induced global change, such as climate change, is altering some hazard parameters of these disasters. These alterations can have serious consequences for vulnerable populations, which often experience post-disaster infectious disease outbreaks, leading to morbidity and mortality. The risks and drivers for these outbreaks are somewhat contested, with human population displacement currently suggested as an important factor, due to its ability to form risk cascades. The globally displaced population is significant, with large numbers of people newly displaced every year. Despite this, reviews quantifying disaster-related disease outbreaks have been on short time scales, specific geographic areas or specific hazards. This review aims to fill this gap and gain a greater understanding of the risk factors involved in these contextual outbreaks on a global level.
Methods and analysis
Using the PRISMA-P 2015 checklist and Khan's methodological framework, a systematic search strategy will be created to find relevant literature in MEDLINE, Embase and GlobalHealth electronic databases, along with the screening of review and selected literature reference lists. Predetermined inclusion and exclusion criteria will be used by a reviewer (GC) to determine eligibility. Studies will not be excluded based on geographic area or publication date, but studies without an English abstract or full-text will be. Following selection, data will be extracted into a data charting form, that will be reviewed by other members of the team. The data will then be analysed both numerically and narratively.
Ethics and dissemination
Only secondary data will be used and therefore no ethical approval is needed. Our findings will aim to be disseminated through a peer-reviewed journal. The authors intend to use the results to inform future mathematical modelling studies.

Strengths and limitations of this study
- The lack of temporal or geographic limits is a major strength of this study, as it allows the authors and readers to gain a global understanding.
- Studies identified will be subject to a quality appraisal and only certain publication methods will be selected, making the studies more comparable.
- A limitation of this study is excluding papers without an abstract or full-text in English, as this creates publication bias.
- A potentially important source of information may be missed by limiting the papers to outbreaks affecting local populations only, removing outbreaks in refugee camps.
- This review will only search peer-reviewed literature and therefore outbreaks that were reported outside the academic literature will not be included.

INTRODUCTION
Disasters have many forms, including those related to natural hazards (e.g., earthquakes, volcanic eruptions, storm surges, floods, droughts, heatwaves, tsunamis) and armed conflict (e.g., terrorism, civil war, genocide, political riots).[1-2] Disaster monitoring systems have shown alterations in frequency and intensity trends.[3-4] A possible contribution to this is human-induced global change, such as climate change, which is altering some hazard parameters.[5-6] For example:

For peer review only

- Natural hazards - Sea level rise and warming temperatures may alter hurricane frequency and intensity.[7-8]
- Armed conflict - Increased temperatures may alter drought frequency, potentially influencing conflict escalation.[9-10]

The potential for these trends to alter the vulnerability of disaster-affected populations demonstrates the importance of understanding the complexities of how disasters impact people. The Sendai Framework for Disaster Risk Reduction[11] states that understanding disaster risk is the number one priority and risk management strategies should protect people and their property, health, livelihoods and productive assets while promoting and protecting all human rights. Understanding and managing risks posed by disasters is therefore paramount to reaching the Sustainable Development Goals.[12] A way in which disasters impact human health is via infectious disease outbreaks, which have been extensively reported.[13-16]

Across diseases, outbreaks have a variety of influences (e.g., climate, food, water, sanitation, health systems) and can be exacerbated by complex interactions among such factors.[17] Understanding these causes is important for anticipating future disease risks in a rapidly changing world. After a disaster, the risk of infectious disease is low but real; for example, an outbreak of norovirus in Texas after Hurricane Katrina in 2005[18] and cutaneous leishmaniasis outbreaks beginning in 2013, during the Syrian conflict.[19] These outbreaks have a significant social and economic cost to already fractured communities and often show inequalities and insufficiencies that were present long before the outbreak or disaster. These issues show the complexity of outbreak risks in this context and the importance of identifying them.[20] A significant risk suggested for these outbreaks is mass displacement.[16, 21-24]

Mass displacement can exacerbates insecurity, potentially leading to further displacement and breakdown of healthcare, services and infrastructure, triggering risk factor cascades, an example of which is shown in Figure 1.[25] The consideration of these factors is very important in understanding population risk (risk = hazard x vulnerability), with exposure sometimes added as another term to multiply, especially when used in epidemiological contexts. Understanding all these factors is important as over-emphasis on a single hazard (such as a natural hazard or armed conflict), reduces the understanding of population exposure and vulnerability.[26] The population of people forcibly displaced, increased from 43.3 million in 2009 to 70.8 million in 2018.[27]; in line with an increased global population of 6.8 to 7.6 billion, in the same time period.[28] Large numbers of people are displaced by natural hazards or armed conflicts; for example, in 2018 natural hazards and armed conflicts contributing to 17.2 million and 10.8 million displaced people, respectively.[29]

The intersection of disasters and displacement therefore provides a unique angle to understand the mechanisms through which climate change can yield health impacts.[30-31] Alterations in disaster parameters and a large displaced population means that understanding the risks of disease outbreaks is crucial to preventing morbidity and mortality. Previous research that has attempted to collate individual disaster-related disease outbreaks has previously been on relatively short time scales,[23] specific geographic areas,[32] or focused on a certain hazard.[33] This review aims to address this gap, to gain a global overview of these outbreaks and highlight potential areas where post-disaster relief needs to focus to reduce disease risk.

AIMS AND OBJECTIVES

To identify disaster-related disease outbreaks and the risk factors that lead to these outbreaks via a comprehensive search of current literature. This will enable understanding of common etiological agents in disaster-related outbreaks, along with regions which are frequently impacted.

Objectives of this review are:

1. Provide a global overview of infectious disease outbreaks that occurred in a post-disaster (natural hazard or armed conflict) setting.
2. Understand the risk factors that lead to these outbreaks and how they link to form cascades.
3. Quantify which geographic areas and etiological agents commonly impact disaster-affected communities.
4. Breakdown disaster type to understand if this impacts the etiological agent causing the outbreak.
5. Suggest areas where global change, such as climate change, may exacerbate the identified risk factors.

6. Disseminate the review findings on global disaster-related disease outbreaks.

METHODS

Systematic reviews are commonly used for evidence-based public health research, both for clinical practice and biomedical research. They sit at the top of the hierarchy of evidence for medical research, as they are considered highly filtered research with low levels of bias.[34] For reviews to be classified as systematic they must first formulate questions, then appraise relevant studies on their quality and finally summarise the evidence found.[35] Indication in this case for a systematic review includes; (1) uncovering the international evidence, (2) identifying areas for further research and (3) investigating conflicting results.[36] An aetiology or risk systematic review most closely matches the review proposed here and is used to determine whether and to what degree a relationship exists between an exposure and a health outcome. For this review, the question needs to outline the exposure, disease and health condition of interest, the population and its location and the study period where relevant. The types of studies included will mainly involve observational studies, namely retrospective, prospective, cross-sectional, case-control and cohort studies.[36-37]

Protocol design

The PRISMA-P 2015 checklist (see appendix 1) will be used to develop the review protocol and will be guided by the methodological approach delineated by Khan *et al.*[38] The framework is set out to follow five stages; (1) framing the question(s), (2) identifying relevant work, (3) assessing study quality, (4) summarising the evidence and (5) interpreting the findings.

Stage 1: Framing the research questions

After preliminary research on natural hazards and armed conflicts and their risk factors for communicable disease outbreaks, it became apparent that displacement was an important factor. It also became clear that quantification of these contextual outbreaks and their risks was insufficient to gain a clear global understanding of the issue. Due to this deficiency, the review questions are as follows (Table 1):

**Table 1: Research questions**
- How important is human displacement as a risk factor cascade in causing disaster-associated community-acquired disease outbreaks?
- What other risk factors are important in causing these outbreaks and how are they potentially linked to form cascades?
- Which pathogens, disasters, global changes including climate change and geographic areas are commonly implicated in outbreaks in a post-disaster setting?

Stage 2: Identifying relevant work

Search strategy

The following electronic databases will be searched; MEDLINE, Embase and Global Health but no grey literature will be used. Reference lists of selected papers and reviews will also be screened for relevant papers. Following the development of both key and MeSH terms, the strategy will be reviewed by the Imperial College medical librarian. Terms will vary depending on the database and will be related to; (1) natural hazards, (2) armed conflict and (3) infectious disease outbreaks (see appendix 2). No standard definitions for natural hazards, armed conflicts and disease outbreaks will be set, as this may exclude important studies, along with any specific risk factors. No temporal or geographic limits will be set and no specific risk factors will be searched to avoid bias in the search results. Additional searches may be requested later to identify any publications potentially missed. Results will be imported into Zotero reference management software.

Along with broad terms for outbreaks, specific diseases will also be searched as identified by the World Health Organisation as common communicable disease outbreaks following disasters,[39] along with commonly reported diseases identified from preliminary scoping searches. Specific pathogens will include those capable of causing acute outbreaks but not causing an outbreak before the disaster. Therefore, despite evidence for contextual increases, [40-41] HIV, hepatitis B, hepatitis C and tuberculosis will not be searched, as they often cause more chronic disease and have a wide range of social implications beyond the scope of this study. Soft tissue, wound infections, inhaled fungal spores and aspiration pneumonia (tsunami lung) will

For peer review only

also not be included, as such infections would only impact those that had open wounds and exposure to the pathogen in the environment. Therefore, it would not cause a widespread outbreak within the population.

Stage 3: Assessing study quality
After the removal of duplicates using Zotero software, search results will be screened. The aim will be to assess the study quality and decide on selection by comparing the publication against eligibility criteria (Table 2). There are >100 critical appraisal tools published and selecting the most useful is challenging; as there is no 'gold standard' for any study design or a widely accepted generic tool.[42] After consideration of published tools, the OHAT risk of bias tool and NIH study quality assessment tools will be used. The NIH tool was chosen as it best captures the range of studies this research aims to review.[43-44] The papers will be screened by one reviewer (GC) and ineligible papers eliminated. All titles and abstracts that meet the criteria will be subjected to full-text reading. A PRISMA flow diagram will then be used, to show the number of publications selected and increase transparency.

Table 2: Inclusion criteria

Population	- Any local population/community impacted by a disaster-related disease outbreak.
Intervention	- Any investigation carried out to quantify a disease outbreak and understand the risk factors.
Comparator	- Members of the disaster-affected population who did not acquire an infection during the outbreak.
Outcomes	- The primary outcome is to understand disaster-related disease outbreaks on a global scale.<br>- The secondary outcome consists of identifying the risk factors that result in these outbreaks.
Study type	- Either retrospective or prospective observational studies, namely, cross-sectional, case-control and cohort studies.<br>- Full-text or abstracts in English

Exclusion criteria
- Papers without an explicit link between a disaster and an outbreak.
- Outbreaks in refugees/populations internationally displaced or in foreign armed forces or aid workers.
- Non-English abstract and full-text publications.
- Other review papers.
- Single case reports.
- Publications discussing general risk factors, public health, mental health and other non-communicable diseases, pathogen genetics or economic costs in a post-disaster setting.

Stage 4: Summarising the evidence
A predetermined data charting form will be used based on preliminary reading and the objectives of the review. Extracted data will include information on the publication (title, authors, date, journal), disaster type, disease, case numbers, study area, study period, identified risk factors, methodological details (study design, sample sizes, laboratory tests, statistical analysis) along with any other relevant data. To ensure all relevant data are collected, the form will be reviewed by other members of the research team before implementation and the data will be extracted independently by the reviewer (GC).

Stage 5: Interpreting the findings
Following the data extraction and to help illustrate how the information collected answers the aims and objectives, the results will be presented both; (1) numerically, with outbreaks broken down and quantified by geographic region, pathogen type and disaster type, along with statistical analysis to understand the importance of risk factors and (2) narratively, by synthesising the methods used, the importance of global change and the links between risk factors and possible cascades. As this is ongoing research, with no data currently collected, details of this stage remain in progress and will be finalised by June 2020. The ability to conduct a meta-analysis will depend on the volume and quality of literature that is returned from the literature searches and therefore will be decided after the data collection stage.

For peer review only

ETHICS AND DISSEMINATION

Following review completion, a global overview of disaster-related disease outbreaks will be determined through time. To assess whether the research aims have been met, the authors will use the GRADE (Grading of Recommendations, Assessment, Development and Evaluations) framework.[45] If the aims are met, the study findings will allow the authors to determine important risk factors for these outbreaks and how they form cascades and links. It will also help form greater understanding of the observational methods used and how effective these have been in capturing and presenting these outbreaks. The results will determine important areas for future research by providing a greater understanding of how different geographic area and hazards impact disease outbreaks. These findings will help to inform later quantitative studies into the dynamics of post-disaster outbreaks, by understand areas where further research is needed, and the best data is available.

As data collection for systematic reviews uses exclusively secondary data, no ethical approval is needed. The aim is to disseminate the review findings through publication in a peer-reviewed journal. Understanding how outbreaks impact vulnerable post-disaster populations and how under global changes this may be altered is very important in preventing mortality and morbidity. It is essential that the rights of these populations are protected and therefore their ability to protect their health in humanitarian crisis. We aim to use the knowledge from this review to guide future computational modelling studies into how displacement and its associated risk factors initiate and spread disease during post-disaster outbreaks.

REFERENCES

[1] Eshghi K, Larson R. Disasters: lessons from the past 105 years. *Disaster Prevention and Management* 2008;17:62-82.

[2] Shaluf IM. An overview on disasters. *Disaster Prevention and Management* 2007;16:687-703.

[3] Dupuy K, Rustad SA. Trends in Armed Conflict, 1946–2017. *Conflict Trends* 2018;5:2018.

[4] Munich Re. NatCatSERVICE. https://natcatservice.munichre.com (accessed 19 Dec 2019).

[5] Coumou D, Rahmstorf S. A decade of weather extremes. *Nature climate change* 2012;2:491-496.

[6] Kelman I. Climate change and the Sendai framework for disaster risk reduction. *International Journal of Disaster Risk Science* 2015;6:117-127.

[7] Below R, Wallemacq P. Annual disaster statistical review 2017. Brussels, Belgium: Centre for Research on the Epidemiology of Disasters 2018.

[8] Stocker TF, Qin D, Plattner GK, *et al.* Climate change 2013: The physical science basis. Contribution of working group I to the fifth assessment report of the intergovernmental panel on climate change. Cambridge, United Kingdom: Cambridge University Press 2013:1535.

[9] Kelley CP, Mohtadi S, Cane MA, *et al*. Climate change in the Fertile Crescent and implications of the recent Syrian drought. *Proceedings of the National Academy of Sciences* 2015;112:3241-3246.

[10] Hsiang SM, Meng KC, Cane MA. Civil conflicts are associated with the global climate. *Nature* 2011;476:438.

[11] United Nations International Strategy for Disaster Reduction. Sendai Framework for Disaster Risk Reduction 2015-2030. New York, USA; United Nations 2015.

[12] United Nations. Sustainable development goals. 17 goals to transform our world. https://www.un.org/sustainabledevelopment/ (accessed 14 Apr 2020).

[13] Al-shere TA, Ujiie M, Suzuki M, *et al.* Outbreak of leptospirosis after flood, the Philippines, 2009. *Emerging infectious diseases* 2012;18:91.

For peer review only

[14] Bompangue D, Giraudoux P, Piarroux M, *et al.* Cholera epidemics, war and disasters around Goma and Lake Kivu: an eight-year survey. *PLoS neglected tropical diseases* 2009;3:e436.

[15] Walton DA, Ivers LC. Responding to cholera in post-earthquake Haiti. *New England Journal of Medicine* 2011;364:3-5.

[16] Watson JT, Gayer M, Connolly MA. Epidemics after natural disasters. *Emerging infectious diseases* 2007;13:1.

[17] Cohen ML. Changing patterns of infectious disease. *Nature* 2000;406:762.

[18] Yee EL, Palacio H, Atmar RL, *et al.* Widespread outbreak of norovirus gastroenteritis among evacuees of Hurricane Katrina residing in a large "megashelter" in Houston, Texas: lessons learned for prevention. *Clinical Infectious Diseases* 2007;44:1032-1039.

[19] Al-Salem WS, Pigott DM, Subramaniam K,*et al.* Cutaneous leishmaniasis and conflict in Syria. *Emerging infectious diseases* 2016;22:931.

[20] Tappero JW, Tauxe RV. Lessons learned during public health response to cholera epidemic in Haiti and the Dominican Republic. Emerging infectious diseases 2011;17:2087.

[21] Fiasca F, Mattei A, Vittorini P, *et al*. Bacterial Meningitis Hospitalizations after the 2009 L'Aquila Earthquake: A Retrospective Observational Study. *Asian J Epidemiol* 2018;11:46-51.

[22] Gayer M, Legros D, Formenty P, *et al.* Conflict and emerging infectious diseases. *Emerging infectious diseases* 2007;13:1625.

[23] Kouadio IK, Aljunid S, Kamigaki T, *et al.* Infectious diseases following natural disasters: prevention and control measures. *Expert review of anti-infective therapy* 2012;10:95-104.

[24] Leaning J, Guha-Sapir D. Natural disasters, armed conflict, and public health. *New England journal of medicine* 2013;369:1836-1842.

[25] Hammer CC, Brainard J, Hunter PR. Risk factors and risk factor cascades for communicable disease outbreaks in complex humanitarian emergencies: A qualitative systematic review. *BMJ global health* 2018;3:e000647.

[26] Cardona OD, van Aalst MK, Birkmann J, *et al.* Determinants of risk: exposure and vulnerability. In: Field CB, Barros V, Stocker TF, *et al,* eds. Managing the Risks of Extreme Events and Disasters to Advance Climate Change Adaptation. A Special Report of Working Groups I and II of the Intergovernmental Panel on Climate Change (IPCC). Cambridge, UK: Cambridge University Press 2012:65-108.

[27] United Nations High Commission for Refugees. Global Trends. Forced Displacement in 2017. Geneva, Switzerland: United Nations High Commissioner for Refugees 2017.

[28] The World Bank. Population, total 2018. https://data.worldbank.org/indicator/sp.pop.totl (accessed 20 Apr 2020).

[29] Anzellini V, Valderrama AC, Desai B, *et al.* Global Report on Internal Displacement 2019. Geneva, Switzerland: International Displacement Monitoring Centre (IDMC) 2019.

[30] Loebach P, Korinek K. Disaster vulnerability, displacement, and infectious disease: Nicaragua and Hurricane Mitch. Population and Environment 2019;40:434-455.

For peer review only

[31] Watts N, Amann M, Arnell N, *et al.* The 2019 report of The Lancet Countdown on health and climate change: ensuring that the health of a child born today is not defined by a changing climate. The Lancet 2019;394:1836-1878.

[32] Suk JE, Vaughan EC, Cook RG, *et al.* Natural disasters and infectious disease in Europe: a literature review to identify cascading risk pathways. European journal of public health 2019.

[33] Wilder-Smith A. Tsunami in South Asia: what is the risk of post-disaster infectious disease outbreaks?. Annals-Academy of Medicine Singapore 2005;34:625.

[34] Evans D. Hierarchy of evidence: a framework for ranking evidence evaluating healthcare interventions. Journal of clinical nursing 2003;12:77-84.

[35] Munn Z, Peters MD, Stern C, *et al.* Systematic review or scoping review? Guidance for authors when choosing between a systematic or scoping review approach. BMC medical research methodology 2018;18:143.

[36] Moola S, Munn Z, Sears K, *et al.* Conducting systematic reviews of association (etiology): The Joanna Briggs Institute's approach. International journal of evidence-based healthcare 2015;13: 163-169.

[37] Munn Z, Stern C, Aromataris E, *et al.* What kind of systematic review should I conduct? A proposed typology and guidance for systematic reviewers in the medical and health sciences. BMC medical research methodology 2018;18:5.

[38] Khan KS, Kunz R, Kleijnen J, *et al.* Five steps to conducting a systematic review. Journal of the royal society of medicine 2003;96:118-121.

[39] World Health Organisation. Communicable diseases following natural disasters. Risk assessment and priority interventions. Geneva, Switzerland: World Health Organisation 2006.

[40] Khan S, Rai MA, Khan A, *et al.* Prevalence of HCV and HIV infections in 2005-Earthquake-affected areas of Pakistan. BMC infectious diseases 2008;8:147.

[41] Kimbrough W, Saliba V, Dahab M, *et al.* The burden of tuberculosis in crisis-affected populations: a systematic review. The Lancet infectious diseases 2012;12:950-965.

[42] Katrak P, Bialocerkowski AE, Massy-Westropp N, *et al.* A systematic review of the content of critical appraisal tools. BMC medical research methodology 2004;4:22.

[43] OHAT. OHAT Risk of Bias Rating Tool for Human and Animal Studies. 2015. https://ntp.niehs.nih.gov/ntp/ohat/pubs/riskofbiastool_508.pdf (accessed 18 Apr 2020).

[44] National Institute of Health. National Health, Lung and Blood Institute. Study Quality Assessment Tools. https://www.nhlbi.nih.gov/health-topics/study-quality-assessment-tools (accessed 18 Apr 2020).

[45] Guyatt G, Oxman AD, Akl EA, Kunz R, Vist G, Brozek J, et al. GRADE guidelines: 1. Introduction-GRADE evidence profiles and summary of findings tables. Journal of clinical epidemiology. 2011;64(4):383-94.

Acknowledgements GECC, KG, KAM and IL acknowledge joint Centre funding from the UK Medical Research Council and Department for International Development [MR/R015600/1]. We would also like to thank the Imperial College medical librarian for his advice on the systematic search strategy.

Author's contributions GC conducted preliminary research and searches, devised the search strategy and wrote the report, incorporated feedback and finalised the manuscript. KG revised the first draft and provided feedback based on her infectious disease expertise. IK revised the first draft and provided feedback based on

For peer review only

his disaster and health expertise. KM provided supervision for the research activity planning, assisting in the conceptualisation of ideas and the overarching research goals and aims, along with providing feedback on the first draft.

Funding statement This work was supported by the National Environmental Research Council as part of the Grantham Institute for Climate Change and the Environment's (Imperial College London) Science and Solutions for a Changing Planet Doctoral Training Partnership [NE/S007415/1].

Competing interests statement None declared

Figure 1: Human population displacement as a possible risk factor cascade. ARI; Acute Respiratory Infections. ADI; Acute Diarrheal Infection. WASH; Water, sanitation & hygiene.[Adapted from: 23, 25]

For peer review only

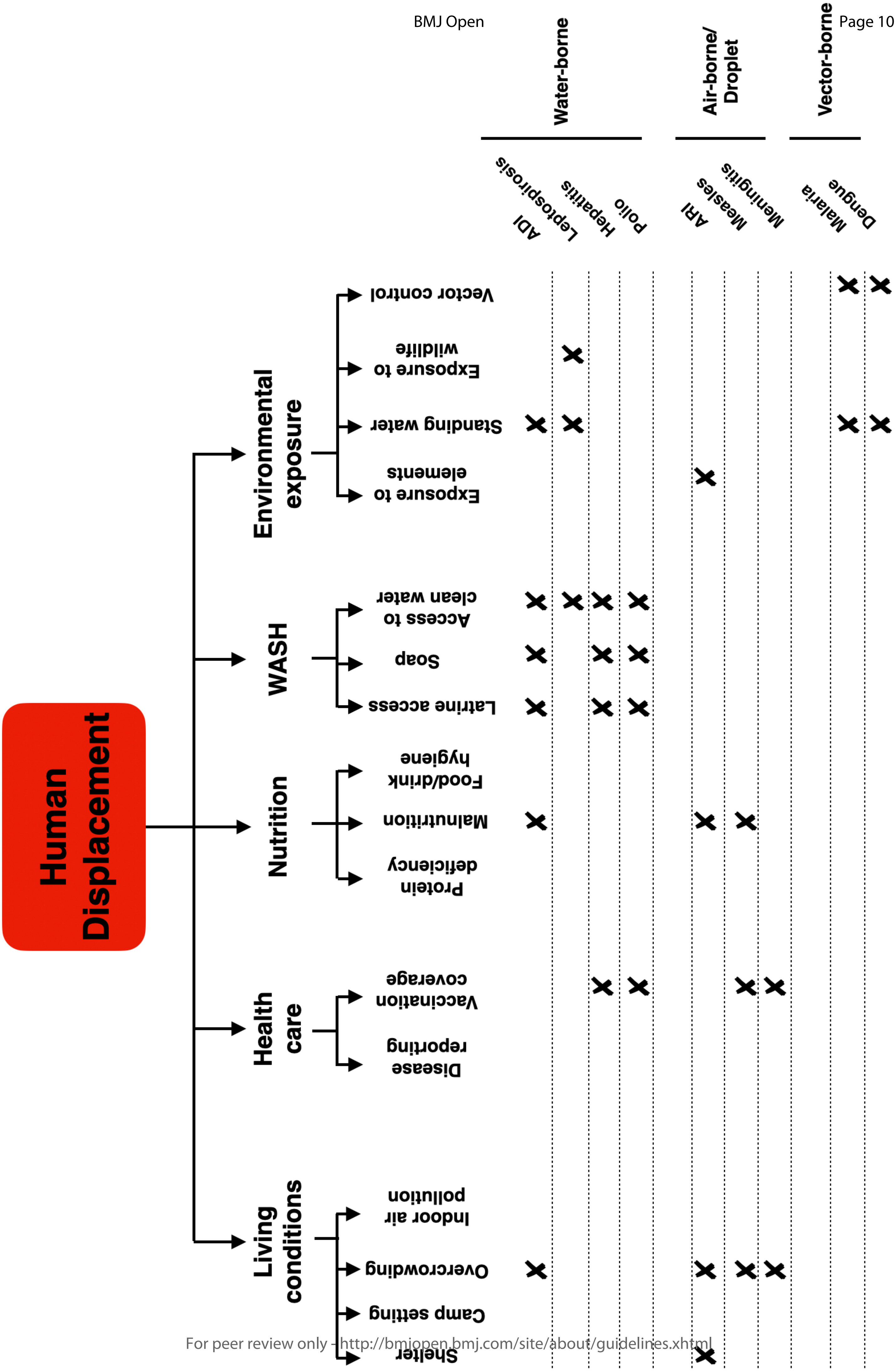
Human Displacement
Environmental exposure
WASH
Nutrition
Health care
Living conditions
Vector control
Exposure to wildlife
Standing water
Exposure to elements
Access to clean water
Soap
Latrine access
Food/drink hygiene
Malnutrition
Protein deficiency
Vaccination coverage
Disease reporting
Indoor air pollution
Overcrowding
Camp setting
Shelter
Water-borne
Air-borne/ Droplet
Vector-borne
ADI
Leptospirosis
Hepatitis
Polio
ARI
Measles
Meningitis
Malaria
Dengue

APPENDICES

Appendix 1 - PRISMA-P (Preferred Reporting Items for Systematic review and Meta-Analysis Protocols) 2015 checklist: recommended items to address in a systematic review protocol

PRISMA-P (Preferred Reporting Items for Systematic review and Meta-Analysis Protocols) 2015 checklist: recommended items to address in a systematic review protocol*

Section and topic	Item No	Checklist item
**ADMINISTRATIVE INFORMATION**		
Title:		
Identification	1a	Identify the report as a protocol of a systematic review
Update	1b	If the protocol is for an update of a previous systematic review, identify as such
Registration	2	If registered, provide the name of the registry (such as PROSPERO) and registration number
Authors:		
Contact	3a	Provide name, institutional affiliation, e-mail address of all protocol authors; provide physical mailing address of corresponding author
Contributions	3b	Describe contributions of protocol authors and identify the guarantor of the review
Amendments	4	If the protocol represents an amendment of a previously completed or published protocol, identify as such and list changes; otherwise, state plan for documenting important protocol amendments
Support:		
Sources	5a	Indicate sources of financial or other support for the review
Sponsor	5b	Provide name for the review funder and/or sponsor
Role of sponsor or funder	5c	Describe roles of funder(s), sponsor(s), and/or institution(s), if any, in developing the protocol
**INTRODUCTION**		
Rationale	6	Describe the rationale for the review in the context of what is already known
Objectives	7	Provide an explicit statement of the question(s) the review will address with reference to participants, interventions, comparators, and outcomes (PICO)
**METHODS**		
Eligibility criteria	8	Specify the study characteristics (such as PICO, study design, setting, time frame) and report characteristics (such as years considered, language, publication status) to be used as criteria for eligibility for the review
Information sources	9	Describe all intended information sources (such as electronic databases, contact with study authors, trial registers or other grey literature sources) with planned dates of coverage
Search strategy	10	Present draft of search strategy to be used for at least one electronic database, including planned limits, such that it could be repeated
Study records:		
Data management	11a	Describe the mechanism(s) that will be used to manage records and data throughout the review
Selection process	11b	State the process that will be used for selecting studies (such as two independent reviewers) through each phase of the review (that is, screening, eligibility and inclusion in meta-analysis)
Data collection process	11c	Describe planned method of extracting data from reports (such as piloting forms, done independently, in duplicate), any processes for obtaining and confirming data from investigators
Data items	12	List and define all variables for which data will be sought (such as PICO items, funding sources), any pre-planned data assumptions and simplifications
Outcomes and prioritization	13	List and define all outcomes for which data will be sought, including prioritization of main and additional outcomes, with rationale
Risk of bias in individual studies	14	Describe anticipated methods for assessing risk of bias of individual studies, including whether this will be done at the outcome or study level, or both; state how this information will be used in data synthesis
Data synthesis	15a	Describe criteria under which study data will be quantitatively synthesised
	15b	If data are appropriate for quantitative synthesis, describe planned summary measures, methods of handling data and methods of combining data from studies, including any planned exploration of consistency (such as I^2, Kendall's τ)
	15c	Describe any proposed additional analyses (such as sensitivity or subgroup analyses, meta-regression)
	15d	If quantitative synthesis is not appropriate, describe the type of summary planned
Meta-bias(es)	16	Specify any planned assessment of meta-bias(es) (such as publication bias across studies, selective reporting within studies)
Confidence in cumulative evidence	17	Describe how the strength of the body of evidence will be assessed (such as GRADE)

*** It is strongly recommended that this checklist be read in conjunction with the PRISMA-P Explanation and Elaboration (cite when available) for important clarification on the items. Amendments to a review protocol should be tracked and dated.** **The copyright for PRISMA-P (including checklist) is held by the PRISMA-P Group and is distributed under a Creative Commons Attribution Licence 4.0.**

Appendix 2 - Search Strategies

Category	Keywords	MeSH MEDLINE	MeSH Embase	MeSH GlobalHealth
**Natural Hazards**	natural hazard* OR natural disaster* OR extreme adj2 event*	climatic process exp, cyclonic storms exp, droughts exp, floods exp, tidal waves exp, geological phenomena exp, avalanches exp, earthquakes exp, landslides exp, tsunamis exp, volcanic eruptions exp, wildfires exp, natural disasters exp	natural disaster exp, disaster victim exp, earthquake exp, drought exp, flooding exp, hurricane exp, tsunami exp, landslide exp, avalanche exp, wildfire exp, volcano exp	natural disaster exp, hurricanes exp, tornados exp, typhoons exp, droughts exp, floods exp, earthquakes exp, landslides exp, avalanches exp, tsunami exp, volcanos exp, wildfire exp
**Conflicts**	armed conflict* or civil war*	ethnic violence exp, exposure to violence exp, armed conflicts exp, war exposure exp	war exposure exp, ethnic conflict exp	conflict exp, war exp, aggression exp, fighting exp
**Disease**	infectious disease outbreak* OR communicable disease outbreak*	disease outbreaks exp, epidemics exp, communicable diseases exp, diarrhoea, vibrio infections exp, cholera exp, salmonella infections exp, typhoid fever exp, paratyphoid fever exp, leptospirosis exp, Weil disease, measles exp, measles virus exp, meningitis, bacterial exp, meningitis, escherichia coli exp, meningitis, meaningococcal exp, meningitis, pneumococcal exp, respiratory tract infections exp, malaria exp, dengue exp, tetanus exp, clostridium infections exp, haemorrhagic fevers, viral exp, poliomyelitis exp, poliovirus exp, coccidioidomycosis exp, dysentery exp, leishmaniasis, cutaneous exp, leishmaniasis, visceral exp, hepatitis exp, hepatitis a exp, hepatitis e exp	typhoid fever exp, salmonellosis exp, acute hepatitis exp, hepatitis a virus exp, hepatitis e virus exp, hepatitis a exp, hepatitis e exp, leptospirosis exp, measles exp, respiratory tract infection exp, malaria exp, dengue exp, tetanus exp, coccidioidomycosis exp, haemorrhagic fever exp, poliomyelitis exp, dysentery exp, acute diarrhoea exp, meningitis exp, skin leishmaniasis exp, visceral leishmaniasis exp	outbreaks exp, epidemics exp, infectious diseases exp, diarrhoea exp, cholera exp, vibrio cholerae exp, salmonella typhi exp, typhoid exp, salmonella paratyphi exp, paratyphoid exp, hepatitis a exp, hepatovirus a exp, hepatitis e exp, hepatovirus e exp, leptospirosis exp, measles exp, meningitis exp, bacterial meningitis exp, viral meningitis exp, respiratory diseases exp, malaria exp, dengue exp, tetanus exp, coccidioidomycosis exp, leishmaniasis exp, cutaneous leishmaniasis exp, visceral leishmaniasis exp, haemorrhagic fever exp, poliomyelitis exp, dysentery exp

Reporting checklist for protocol of a systematic review.

Based on the PRISMA-P guidelines.

Instructions to authors

Complete this checklist by entering the page numbers from your manuscript where readers will find each of the items listed below.

Your article may not currently address all the items on the checklist. Please modify your text to include the missing information. If you are certain that an item does not apply, please write "n/a" and provide a short explanation.

Upload your completed checklist as an extra file when you submit to a journal.

In your methods section, say that you used the PRISMA-Preporting guidelines, and cite them as:

Moher D, Shamseer L, Clarke M, Ghersi D, Liberati A, Petticrew M, Shekelle P, Stewart LA. Preferred Reporting Items for Systematic Review and Meta-Analysis Protocols (PRISMA-P) 2015 statement. Syst Rev. 2015;4(1):1.

		Reporting Item	Page Number
**Title**			
Identification	#1a	Identify the report as a protocol of a systematic review	1
Update	#1b	If the protocol is for an update of a previous systematic review, identify as such	n/a
**Registration**			
	#2	If registered, provide the name of the registry (such as PROSPERO) and registration number	n/a
**Authors**			
Contact	#3a	Provide name, institutional affiliation, e-mail address of all protocol authors; provide physical mailing address of corresponding author	1

For peer review only

Contribution	#3b	Describe contributions of protocol authors and identify the guarantor of the review	1
**Amendments**			
	#4	If the protocol represents an amendment of a previously completed or published protocol, identify as such and list changes; otherwise, state plan for documenting important protocol amendments	n/a
**Support**			
Sources	#5a	Indicate sources of financial or other support for the review	8
Sponsor	#5b	Provide name for the review funder and / or sponsor	8
Role of sponsor or funder	#5c	Describe roles of funder(s), sponsor(s), and / or institution(s), if any, in developing the protocol	8
**Introduction**			
Rationale	#6	Describe the rationale for the review in the context of what is already known	2
Objectives	#7	Provide an explicit statement of the question(s) the review will address with reference to participants, interventions, comparators, and outcomes (PICO)	3
**Methods**			
Eligibility criteria	#8	Specify the study characteristics (such as PICO, study design, setting, time frame) and report characteristics (such as years considered, language, publication status) to be used as criteria for eligibility for the review	4
Information sources	#9	Describe all intended information sources (such as electronic databases, contact with study authors, trial registers or other grey literature sources) with planned dates of coverage	4
Search strategy	#10	Present draft of search strategy to be used for at least one electronic database, including planned limits, such that it could be repeated	appendix 2

For peer review only

Study records - data management	#11a	Describe the mechanism(s) that will be used to manage records and data throughout the review	4
Study records - selection process	#11b	State the process that will be used for selecting studies (such as two independent reviewers) through each phase of the review (that is, screening, eligibility and inclusion in meta-analysis)	4
Study records - data collection process	#11c	Describe planned method of extracting data from reports (such as piloting forms, done independently, in duplicate), any processes for obtaining and confirming data from investigators	5
Data items	#12	List and define all variables for which data will be sought (such as PICO items, funding sources), any pre-planned data assumptions and simplifications	4
Outcomes and prioritization	#13	List and define all outcomes for which data will be sought, including prioritization of main and additional outcomes, with rationale	5
Risk of bias in individual studies	#14	Describe anticipated methods for assessing risk of bias of individual studies, including whether this will be done at the outcome or study level, or both; state how this information will be used in data synthesis	4
Data synthesis	#15a	Describe criteria under which study data will be quantitatively synthesised	5
Data synthesis	#15b	If data are appropriate for quantitative synthesis, describe planned summary measures, methods of handling data and methods of combining data from studies, including any planned exploration of consistency (such as I2, Kendall's τ)	5
Data synthesis	#15c	Describe any proposed additional analyses (such as sensitivity or subgroup analyses, meta-regression)	5
Data synthesis	#15d	If quantitative synthesis is not appropriate, describe the type of summary planned	5
Meta-bias(es)	#16	Specify any planned assessment of meta-bias(es) (such as publication bias across studies, selective reporting within	5

For peer review only

		studies)	
Confidence in cumulative evidence	#17	Describe how the strength of the body of evidence will be assessed (such as GRADE)	5

The PRISMA-P checklist is distributed under the terms of the Creative Commons Attribution License CC-BY 4.0. This checklist was completed on 20. April 2020 using https://www.goodreports.org/, a tool made by the EQUATOR Network in collaboration with Penelope.ai

For peer review only

BMJ Open

Understanding the risks for post-disaster infectious disease outbreaks: a systematic review protocol

Journal:	*BMJ Open*
Manuscript ID	bmjopen-2020-039608.R1
Article Type:	Protocol
Date Submitted by the Author:	11-Aug-2020
Complete List of Authors:	Charnley, Gina; Imperial College London Faculty of Medicine, School of Public Health, Department of Infectious Disease Epidemiology<br>Gaythorpe, Katy; Imperial College London Faculty of Medicine, School of Public Health, Department of Infectious Disease Epidemiology<br>Murray, Kris; Imperial College London Faculty of Medicine, School of Public Health, Department of Infectious Disease Epidemiology<br>Kelman, Ilan; University College London Faculty of Mathematical and Physical Sciences, UCL Institute for Risk and Disaster Reduction; University College London Faculty of Population Health Sciences, UCL Institute for Global Health
**Primary Subject Heading**:	Epidemiology
Secondary Subject Heading:	Infectious diseases, Public health, Research methods
Keywords:	Protocols & guidelines < HEALTH SERVICES ADMINISTRATION & MANAGEMENT, Epidemiology < INFECTIOUS DISEASES, PUBLIC HEALTH, Epidemiology < TROPICAL MEDICINE

SCHOLARONE™
Manuscripts

BMJ

I, the Submitting Author has the right to grant and does grant on behalf of all authors of the Work (as defined in the below author licence), an exclusive licence and/or a non-exclusive licence for contributions from authors who are: i) UK Crown employees; ii) where BMJ has agreed a CC-BY licence shall apply, and/or iii) in accordance with the terms applicable for US Federal Government officers or employees acting as part of their official duties; on a worldwide, perpetual, irrevocable, royalty-free basis to BMJ Publishing Group Ltd ("BMJ") its licensees and where the relevant Journal is co-owned by BMJ to the co-owners of the Journal, to publish the Work in this journal and any other BMJ products and to exploit all rights, as set out in our licence.

The Submitting Author accepts and understands that any supply made under these terms is made by BMJ to the Submitting Author unless you are acting as an employee on behalf of your employer or a postgraduate student of an affiliated institution which is paying any applicable article publishing charge ("APC") for Open Access articles. Where the Submitting Author wishes to make the Work available on an Open Access basis (and intends to pay the relevant APC), the terms of reuse of such Open Access shall be governed by a Creative Commons licence – details of these licences and which Creative Commons licence will apply to this Work are set out in our licence referred to above.

Other than as permitted in any relevant BMJ Author's Self Archiving Policies, I confirm this Work has not been accepted for publication elsewhere, is not being considered for publication elsewhere and does not duplicate material already published. I confirm all authors consent to publication of this Work and authorise the granting of this licence.

For peer review only

Understanding the risks for post-disaster infectious disease outbreaks: a systematic review protocol

Gina E. C. Charnley[1], Ilan Kelman[234], Katy A. M. Gaythorpe[1], Kris A. Murray[1]
[1]Faculty of Medicine, School of Public Health, Imperial College London, London, UK. [2]Faculty of Mathematical and Physical Sciences, UCL Institute for Risk and Disaster Reduction & [3]Faculty of Population Health Sciences, UCL Institute for Global Health, University College London, London, UK. [4]University of Agder, Kristiansand, Norway.
Correspondence to Gina Charnley; g.charnley19@imperial.ac.uk; School of Public Health, St. Mary's Campus, Imperial College London, London, W2 1NY.
Word Count 2,774

ABSTRACT
Introduction
Disasters have many forms, including those related to natural hazards and armed conflict. Human-induced global change, such as climate change, may alter hazard parameters of these disasters. These alterations can have serious consequences for vulnerable populations, which often experience post-disaster infectious disease outbreaks, leading to morbidity and mortality. The risks and drivers for these outbreaks and their ability to form cascades, are somewhat contested. Despite evidence for post-disaster outbreaks, reviews quantifying them have been on short time scales, specific geographic areas or specific hazards. This review aims to fill this gap and gain a greater understanding of the risk factors involved in these contextual outbreaks on a global level.
Methods and analysis
Using the PRISMA-P 2015 checklist and Khan's methodological framework, a systematic search strategy will be created and carried out in August 2020. The strategy will search MEDLINE, Embase and GlobalHealth electronic databases and reference lists of selected literature will also be screened. Eligible studies will include any retrospective cross-sectional, case-control or cohort studies investigating an infectious disease outbreak in a local disaster affected population. Studies will not be excluded based on geographic area or publication date. Excluded papers will include non-English studies, reviews, single case studies and research discussing general risk factors, international refugee camps, public health, mental health and other non-communicable diseases, pathogen genetics or economics. Following selection, data will be extracted into a data charting form, that will be reviewed by other members of the team. The data will then be analysed both numerically and narratively.
Ethics and dissemination
Only secondary data will be used and there will be no public or patient involvement; therefore, no ethical approval is needed. Our findings will aim to be disseminated through a peer-reviewed journal. The authors intend to use the results to inform future mathematical modelling studies.

Strengths and limitations of this study
- The lack of temporal or geographic limits is a major strength of this study, as it allows the authors and readers to gain a global understanding.
- Studies identified will be subject to a quality appraisal and only certain publication methods will be selected, making the studies more comparable.
- A limitation of this study is excluding papers without an abstract or full-text in English, as this creates a language bias.
- This review will not create a complete list of disaster-related disease outbreaks as it will only search peer-reviewed literature, which may have a publication bias.

INTRODUCTION
Disasters have many forms, including those related to natural hazards (e.g., earthquakes, volcanic eruptions, storm surges, floods, droughts, heatwaves, tsunamis) and armed conflict (e.g., terrorism, civil war, genocide, political riots).[1-2] Disaster monitoring systems have shown alterations in frequency and intensity trends.[3-4] A possible contribution for this global change is human-induced climate change, through rising carbon dioxide emissions. This may altering some hazard parameters.[5-6] For example:
- Natural hazards - Sea level rise and warming temperatures may alter hurricane frequency and intensity.[7-8]

For peer review only

- Armed conflict - Increased temperatures may alter drought frequency, potentially influencing conflict escalation.[9-10]

The potential for these trends to alter the vulnerability of disaster-affected populations demonstrates the importance of understanding the complexities of how disasters impact people. The Sendai Framework for Disaster Risk Reduction[11] states that understanding disaster risk is the number one priority and risk management strategies should protect people and their property, health, livelihoods and productive assets while promoting and protecting all human rights. Understanding and managing risks posed by disasters is therefore paramount to reaching the Sustainable Development Goals.[12] A way in which disasters impact human health is via infectious disease outbreaks, which have been extensively reported.[13-16]

Across diseases, outbreaks have a variety of factors capable of influencing initiation, duration and severity (e.g., climate, food, water, sanitation, health systems) and can be exacerbated by complex interactions among such factors.[17] Understanding these causes is important for anticipating future disease risks in a rapidly changing world. After a disaster, the risk of infectious disease is low but real; for example, an outbreak of norovirus in Texas after Hurricane Katrina in 2005[18] and cutaneous leishmaniasis outbreaks beginning in 2013, during the Syrian conflict.[19] These outbreaks have a significant social and economic cost to already fractured communities and often show inequalities and insufficiencies that were present long before the outbreak or disaster. These issues show the complexity of outbreak risks in this context and the importance of identifying them.[20]

Several risk factors can lead to disease outbreaks following disasters, such as poor WASH (water, sanitation and hygiene) conditions,[15] alterations in vector behaviour,[21] issues with housing and shelter,[22] problems obtaining healthcare [23] and mass population displacement.[16,24-26] To add further complexity, few risks act solely to cause an outbreak and risk factors are potentially linked, a concept known as risk factor cascades,[27] an example of which is shown in Figure 1, using displacement as the cascade trigger. Examining these risk factors is very important in understanding population risk (risk = hazard x vulnerability x exposure). Several of these risks have the potential to increase vulnerability and over-emphasis on a single hazard (such as a natural hazard or armed conflict), reduces this understanding.[28]

The intersection of disasters and disease outbreaks therefore provides a unique angle to understand the mechanisms through which global change can yield health impacts.[29-30] Alterations in disaster parameters means that understanding the risks of disease outbreaks is crucial to preventing morbidity and mortality. Previous research that has attempted to collate individual disaster-related disease outbreaks has previously been on relatively short time scales (2000-11),[25] specific geographic areas (Europe),[31] or focused on a certain hazard (tsunami).[32] This review aims to address this gap, to gain a global overview of these outbreaks and highlight potential areas where post-disaster relief needs to focus to reduce disease risk.

AIMS AND OBJECTIVES

To identify disaster-related disease outbreaks and the risk factors that lead to these outbreaks via a comprehensive search of current literature. This will enable understanding of common etiological agents in disaster-related outbreaks, along with regions which are frequently impacted.

Objectives of this review are:

1. Provide a global overview of infectious disease outbreaks that occurred in a post-disaster (natural hazard or armed conflict) setting, to examine common geographic regions, disasters and outbreak aetiologies.
2. Examine the risk factors that lead to these outbreaks and how they link to form cascades.
3. Suggest areas where global change, such as climate change, may exacerbate the identified risk factors.
4. Disseminate the review findings on global disaster-related disease outbreaks.

METHODS

Systematic reviews are commonly used for evidence-based public health research, both for clinical practice and biomedical research. They sit at the top of the hierarchy of evidence for medical research, as they are

For peer review only

considered highly filtered research with low levels of bias.[33] For reviews to be classified as systematic they must first formulate questions, then appraise relevant studies on their quality and finally summarise the evidence found.[34] Indication in this case for a systematic review includes; (1) uncovering the international evidence, (2) identifying areas for further research and (3) investigating conflicting results.[35] An aetiology or risk systematic review most closely matches the review proposed here and is used to determine whether and to what degree a relationship exists between an exposure and a health outcome. For this review, the question needs to outline the exposure, disease and health condition of interest, the population and its location and the study period where relevant. The types of studies included will mainly involve observational studies, namely retrospective, cross-sectional, case-control and cohort studies.[35-36]

Protocol design

The PRISMA-P 2015 checklist (see Appendix 1) will be used to develop the review protocol and will be guided by the methodological approach delineated by Khan *et al.*[37] The framework is set out to follow five stages; (1) framing the question(s), (2) identifying relevant work, (3) assessing study quality, (4) summarising the evidence and (5) interpreting the findings.

Stage 1: Framing the research questions

After preliminary research on natural hazards and armed conflicts and their risk factors for communicable disease outbreaks, it became apparent that quantification of these contextual outbreaks and their risks was insufficient to gain a clear global understanding of the issue. Due to this deficiency, the review questions are as follows (Table 1):

**Table 1: Research questions**
- Which pathogens, disasters, global changes and geographic areas are commonly implicated in outbreaks in a post-disaster setting?
- Which risk factors are important in causing disaster-related disease outbreaks and how are they potentially linked to form cascades?

Stage 2: Identifying relevant work

Search strategy

The following electronic databases will be searched; MEDLINE, Embase and Global Health, as these are appropriate for the subject matter, but no grey literature will be used. Reference lists of selected papers and reviews will also be screened for relevant papers and subject to the same screening process. Following the development of both key and MeSH terms, the strategy and database choice will be reviewed by the Imperial College medical librarian, to ensure all relevant literature will be found. No standard definitions will be set but instead terms will vary depending on the database and will be related to; (1) natural hazards, (2) armed conflict and (3) infectious disease outbreaks. Database-specific terms for natural hazards will be searched to including climatological, hydrological, geophysical, meteorological and armed conflict hazards, with a full search strategy shown in Appendix 2. No temporal or geographic limits will be set and no specific risk factors will be searched to avoid bias in the search results. The systematic search will be carried out in August 2020 and additional searches may be requested later to identify any publications potentially missed. Results will be imported into Zotero reference management software.

Along with database-specific broad terms for outbreaks, specific epidemic potential diseases will also be searched, as identified by the World Health Organization as common communicable disease outbreaks following disasters,[38] along with commonly reported diseases identified from preliminary scoping searches. Specific pathogens will include those capable of causing acute outbreaks but not causing an outbreak before the disaster. Therefore, despite evidence for contextual increases,[39-40] HIV, hepatitis B, hepatitis C and tuberculosis will not be searched, as they often cause more chronic disease and have a wide range of social implications beyond the scope of this study. Soft tissue, wound infections, inhaled fungal spores and aspiration pneumonia (tsunami lung) will also not be included, as such infections would only impact those that had open wounds and exposure to the pathogen in the environment. Therefore, they are not considered to have epidemic potential.

Stage 3: Assessing study quality

After the removal of duplicates using Zotero software, search results will be screened by one reviewer (GC). The aim will be to assess the study quality and decide on selection by comparing the publication against

For peer review only

eligibility criteria (Table 2). There are >100 critical appraisal tools published and selecting the most useful is challenging; as there is no 'gold standard' for any study design or a widely accepted generic tool.[41] After consideration of published tools, the NIH study quality assessment tools will be used. The NIH tool was chosen as it best captures the range of studies this research aims to review and accounts for bias and several methodological flaws.[42] A final score of either 'good' (>70% of questions answered 'yes'), 'fair' or 'poor' (<50% answered 'yes') will be given. All studies rating 'poor' will be removed and studies rated 'fair', will be assessed to try and decide if the questions answered 'no' would lead to major bias in answering the research question. One reviewer will be used based on time and personnel constraints of this study and it is acknowledged that this may increase the number of studies missed. Despite this, the authors still believe that the review is systematic as it aims to appraise and synthesis all available literature and provide sufficient detail to be reproducible. All titles and abstracts that meet the criteria will be subjected to full-text reading. A PRISMA flow diagram will then be used, to show the number of publications selected and increase transparency.

**Table 2: Eligibility criteria**	
**Inclusion**	
Population	- Any local population/community impacted by a disaster-related disease outbreak.
Intervention	- Any investigation carried out to quantify a disease outbreak and understand the risk factors.
Comparator	- Members of the disaster-affected population who did not acquire an infection during the outbreak.
Outcomes	- The primary outcome is to understand disaster-related disease outbreaks on a global scale.<br>- The secondary outcome consists of identifying the risk factors that result in these outbreaks.
Study type	- Retrospective observational studies, namely, cross-sectional, case-control and cohort studies.<br>- Full-text or abstracts in English
**Exclusion**	
- Papers without an explicit link between a disaster and an outbreak.	
- Outbreaks in refugees/refugee camps, foreign armed forces, aid workers and international travelers, as this review aims to look at local outbreaks in regional populations.	
- Non-English abstract and full-texts, due to linguistic constraints.	
- Review papers, as only primary sources are desired for this review.	
- Single case reports in diseases not considered to have epidemic potential.	
- Publications discussing general risk factors, public health, mental health and other non-communicable dis pathogen genetics or economic costs in a post-disaster setting, as these are beyond the scope of this reviev its objectives.	

For peer review only

It could be argued that if populations were displaced internationally by a disaster and an outbreak occurred, this may have been due to the disaster. Despite this, international refugee camps commonly housed refugees from multiple countries, impacted by multiple disasters; therefore, linking these outbreaks to specific disasters would be challenging. The only camp settings explored in this review will be national relief camps. By focusing on local populations this will increase the likelihood that the outbreak was caused to the disaster and not due to other external factors.

Stage 4: Summarising the evidence

A predetermined data charting form will be used based on preliminary reading and the objectives of the review. Extracted data will include information on the publication (title, authors, date, journal), disaster type, disease, case numbers, study area, study period, identified risk factors, methodological details (study design, sample sizes, laboratory tests, statistical analysis) along with any other relevant data. To ensure all relevant data are collected, the form will be reviewed by other members of the research team before implementation and the data will be extracted independently by the reviewer (GC).

For peer review only - http://bmjopen.bmj.com/site/about/guidelines.xhtml

Stage 5: Interpreting the findings
Following the data extraction and to help illustrate how the information collected answers the aims and objectives, the results will be presented both; (1) numerically, with outbreaks broken down and quantified by geographic region, pathogen type and disaster type, along with statistical analysis to understand the importance of risk factors and (2) narratively, by synthesising the methods used, the importance of global change and the links between risk factors and possible cascades. As this is ongoing research, with no data currently collected, details of this stage remain in progress and will be finalised by August 2020. The ability to conduct a meta-analysis will depend on the volume and quality of literature that is returned from the searches and therefore will be decided after the data collection stage.

Patient and Public Involvement
Patients and the public were not involved in this research, as the role of this study is to provide a protocol for a review using secondary data.

ETHICS AND DISSEMINATION
Following review completion, a global overview of disaster-related disease outbreaks will be determined through time. To assess whether the research aims have been met, the authors will use the GRADE (Grading of Recommendations, Assessment, Development and Evaluations) framework.[43] If the aims are met, the study findings will allow the authors to determine important risk factors for these outbreaks and how they form cascades and links. It will also help form greater understanding of the observational methods used and how effective these have been in capturing and presenting these outbreaks. The results will determine important areas for future research by providing a greater understanding of how different geographic area and hazards impact disease outbreaks. These findings will help to inform later quantitative studies into the dynamics of post-disaster outbreaks, by understand areas where further research is needed, and the best data is available.

As data collection for systematic reviews uses exclusively secondary data, no ethical approval is needed. The aim is to disseminate the review findings through publication in a peer-reviewed journal. Understanding how outbreaks impact vulnerable post-disaster populations and how under global changes this may be altered is very important in preventing mortality and morbidity. It is essential that the rights of these populations are protected and therefore their ability to protect their health in humanitarian crisis. We aim to use the knowledge from this review to guide future computational modelling studies into how displacement and its associated risk factors initiate and spread disease during post-disaster outbreaks.

REFERENCES
[1] Eshghi K, Larson R. Disasters: lessons from the past 105 years. *Disaster Prevention and Management* 2008;17:62-82.

[2] Shaluf IM. An overview on disasters. *Disaster Prevention and Management* 2007;16:687-703.

[3] Dupuy K, Rustad SA. Trends in Armed Conflict, 1946–2017. *Conflict Trends* 2018;5:2018.

[4] Munich Re. NatCatSERVICE. https://natcatservice.munichre.com (accessed 19 Dec 2019).

[5] Coumou D, Rahmstorf S. A decade of weather extremes. *Nature climate change* 2012;2:491-496.

[6] Kelman I. Climate change and the Sendai framework for disaster risk reduction. *International Journal of Disaster Risk Science* 2015;6:117-127.

[7] Below R, Wallemacq P. Annual disaster statistical review 2017. Brussels, Belgium: Centre for Research on the Epidemiology of Disasters 2018.

[8] Stocker TF, Qin D, Plattner GK, *et al.* Climate change 2013: The physical science basis. Contribution of working group I to the fifth assessment report of the intergovernmental panel on climate change. Cambridge, United Kingdom: Cambridge University Press 2013:1535.

For peer review only

[9] Kelley CP, Mohtadi S, Cane MA, *et al*. Climate change in the Fertile Crescent and implications of the recent Syrian drought. *Proceedings of the National Academy of Sciences* 2015;112:3241-3246.

[10] Hsiang SM, Meng KC, Cane MA. Civil conflicts are associated with the global climate. *Nature* 2011;476:438.

[11] United Nations International Strategy for Disaster Reduction. Sendai Framework for Disaster Risk Reduction 2015-2030. New York, USA; United Nations 2015.

[12] United Nations. Sustainable development goals. 17 goals to transform our world. https://www.un.org/sustainabledevelopment/ (accessed 14 Apr 2020).

[13] Al-shere TA, Ujiie M, Suzuki M, *et al.* Outbreak of leptospirosis after flood, the Philippines, 2009. *Emerging infectious diseases* 2012;18:91.

[14] Bompangue D, Giraudoux P, Piarroux M, *et al.* Cholera epidemics, war and disasters around Goma and Lake Kivu: an eight-year survey. *PLoS neglected tropical diseases* 2009;3:e436.

[15] Walton DA, Ivers LC. Responding to cholera in post-earthquake Haiti. *New England Journal of Medicine* 2011;364:3-5.

[16] Watson JT, Gayer M, Connolly MA. Epidemics after natural disasters. *Emerging infectious diseases* 2007;13:1.

[17] Cohen ML. Changing patterns of infectious disease. *Nature* 2000;406:762.

[18] Yee EL, Palacio H, Atmar RL, *et al.* Widespread outbreak of norovirus gastroenteritis among evacuees of Hurricane Katrina residing in a large "megashelter" in Houston, Texas: lessons learned for prevention. *Clinical Infectious Diseases* 2007;44:1032-1039.

[19] Al-Salem WS, Pigott DM, Subramaniam K,*et al.* Cutaneous leishmaniasis and conflict in Syria. *Emerging infectious diseases* 2016;22:931.

[20] Tappero JW, Tauxe RV. Lessons learned during public health response to cholera epidemic in Haiti and the Dominican Republic. Emerging infectious diseases 2011;17:2087.

[21] 14. Baqir, M. et al. Infectious diseases in the aftermath of monsoon flooding in Pakistan. *Asian Pac. J. Trop. Biomed.* **2,** 76-79 (2012).

[22] Fiasca F, Mattei A, Vittorini P, *et al*. Bacterial Meningitis Hospitalizations after the 2009 L'Aquila Earthquake: A Retrospective Observational Study. *Asian J Epidemiol* 2018;11:46-51.

[23] Valente, F. et al. Massive outbreak of poliomyelitis caused by type-3 wild poliovirus in Angola in 1999. *Bulletin of the World Health Organization* **78,** 339-346 (2000).

[24] Gayer M, Legros D, Formenty P, *et al.* Conflict and emerging infectious diseases. *Emerging infectious diseases* 2007;13:1625.

[25] Kouadio IK, Aljunid S, Kamigaki T, *et al.* Infectious diseases following natural disasters: prevention and control measures. *Expert review of anti-infective therapy* 2012;10:95-104.

[26] Leaning J, Guha-Sapir D. Natural disasters, armed conflict, and public health. *New England journal of medicine* 2013;369:1836-1842.

[27] Hammer CC, Brainard J, Hunter PR. Risk factors and risk factor cascades for communicable disease outbreaks in complex humanitarian emergencies: A qualitative systematic review. *BMJ global health* 2018;3:e000647.

For peer review only

[28] Cardona OD, van Aalst MK, Birkmann J, *et al.* Determinants of risk: exposure and vulnerability. In: Field CB, Barros V, Stocker TF, *et al,* eds. Managing the Risks of Extreme Events and Disasters to Advance Climate Change Adaptation. A Special Report of Working Groups I and II of the Intergovernmental Panel on Climate Change (IPCC). Cambridge, UK: Cambridge University Press 2012:65-108.

[29] Loebach P, Korinek K. Disaster vulnerability, displacement, and infectious disease: Nicaragua and Hurricane Mitch. Population and Environment 2019;40:434-455.

[30] Watts N, Amann M, Arnell N, *et al.* The 2019 report of The Lancet Countdown on health and climate change: ensuring that the health of a child born today is not defined by a changing climate. The Lancet 2019;394:1836-1878.

[31] Suk JE, Vaughan EC, Cook RG, *et al.* Natural disasters and infectious disease in Europe: a literature review to identify cascading risk pathways. European journal of public health 2019.

[32] Wilder-Smith A. Tsunami in South Asia: what is the risk of post-disaster infectious disease outbreaks?. Annals-Academy of Medicine Singapore 2005;34:625.

[33] Evans D. Hierarchy of evidence: a framework for ranking evidence evaluating healthcare interventions. Journal of clinical nursing 2003;12:77-84.

[34] Munn Z, Peters MD, Stern C, *et al.* Systematic review or scoping review? Guidance for authors when choosing between a systematic or scoping review approach. BMC medical research methodology 2018;18:143.

[35] Moola S, Munn Z, Sears K, *et al.* Conducting systematic reviews of association (etiology): The Joanna Briggs Institute's approach. International journal of evidence-based healthcare 2015;13: 163-169.

[36] Munn Z, Stern C, Aromataris E, *et al.* What kind of systematic review should I conduct? A proposed typology and guidance for systematic reviewers in the medical and health sciences. BMC medical research methodology 2018;18:5.

[37] Khan KS, Kunz R, Kleijnen J, *et al*. Five steps to conducting a systematic review. Journal of the royal society of medicine 2003;96:118-121.

[38] World Health Organisation. Communicable diseases following natural disasters. Risk assessment and priority interventions. Geneva, Switzerland: World Health Organisation 2006.

[39] Khan S, Rai MA, Khan A, *et al.* Prevalence of HCV and HIV infections in 2005-Earthquake-affected areas of Pakistan. BMC infectious diseases 2008;8:147.

[40] Kimbrough W, Saliba V, Dahab M, *et al.* The burden of tuberculosis in crisis-affected populations: a systematic review. The Lancet infectious diseases 2012;12:950-965.

[41] Katrak P, Bialocerkowski AE, Massy-Westropp N, *et al.* A systematic review of the content of critical appraisal tools. BMC medical research methodology 2004;4:22.

[42] National Institute of Health. National Health, Lung and Blood Institute. Study Quality Assessment Tools. https://www.nhlbi.nih.gov/health-topics/study-quality-assessment-tools (accessed 18 Apr 2020).

[43] Guyatt G, Oxman AD, Akl EA, Kunz R, Vist G, Brozek J, et al. GRADE guidelines: 1. Introduction-GRADE evidence profiles and summary of findings tables. Journal of clinical epidemiology. 2011;64(4):383-94.

For peer review only

Acknowledgements GECC, KAMG, KAM and IL acknowledge joint Centre funding from the UK Medical Research Council and Department for International Development [MR/R015600/1]. We would also like to thank the Imperial College medical librarian for his advice on the systematic search strategy.

Author's contributions GC conducted preliminary research and searches, devised the search strategy and wrote the report, incorporated feedback and finalised the manuscript. KG revised the first draft and provided feedback based on her infectious disease expertise. IK revised the first draft and provided feedback based on his disaster and health expertise. KM provided supervision for the research activity planning, assisting in the conceptualisation of ideas and the overarching research goals and aims, along with providing feedback on the first draft.

Funding statement This work was supported by the National Environmental Research Council as part of the Grantham Institute for Climate Change and the Environment's (Imperial College London) Science and Solutions for a Changing Planet Doctoral Training Partnership [NE/S007415/1].

Competing interests statement None declared

Figure 1: Human population displacement as a possible risk factor cascade. ARI; Acute Respiratory Infections. ADI; Acute Diarrheal Infection. WASH; Water, sanitation & hygiene.[Adapted from**:** 25, 27]

For peer review only

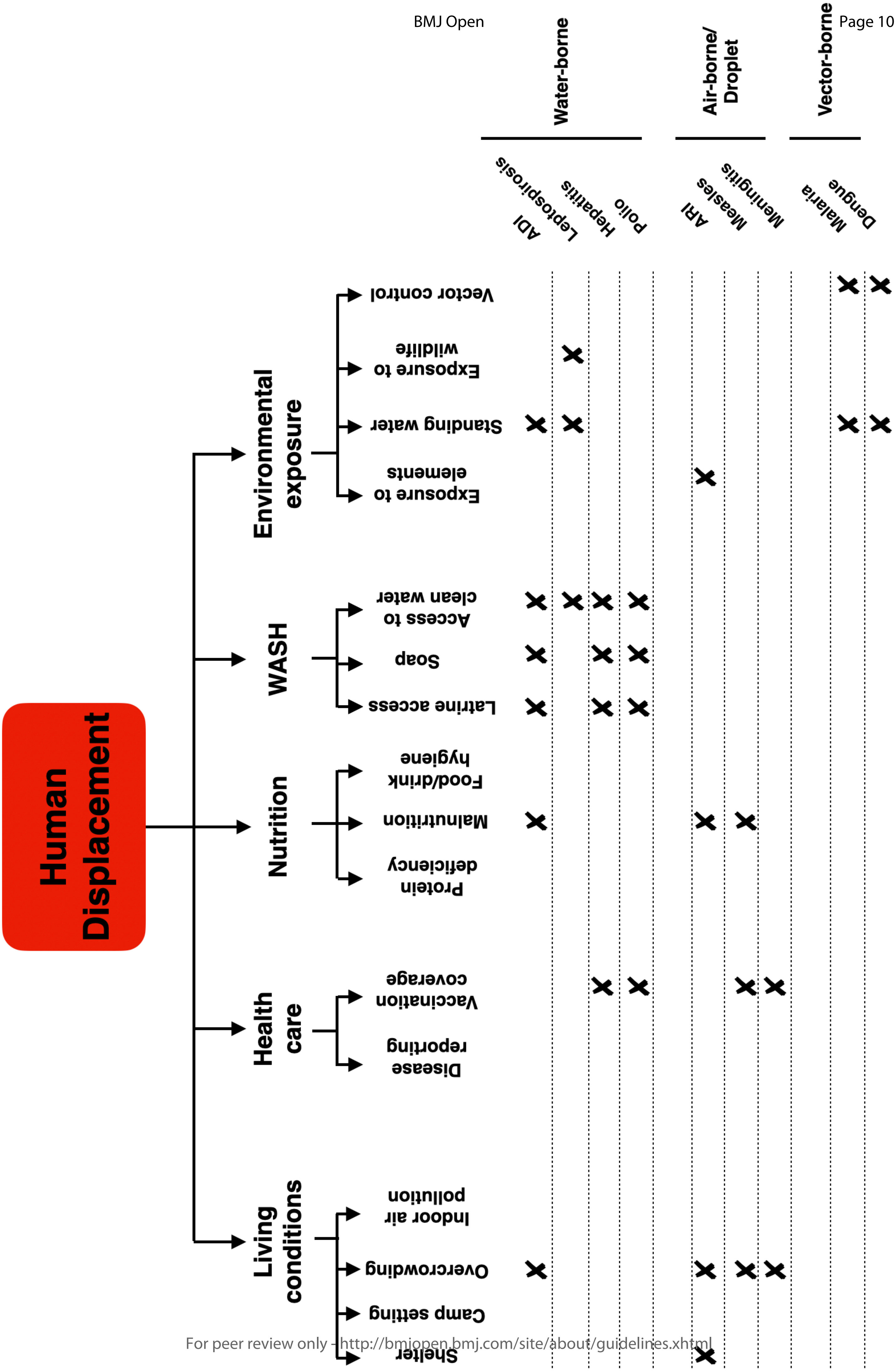

For peer review only - http://bmjopen.bmj.com/site/about/guidelines.xhtml

APPENDICES

Appendix 1 - PRISMA-P (Preferred Reporting Items for Systematic review and Meta-Analysis Protocols) 2015 checklist: recommended items to address in a systematic review protocol

PRISMA-P (Preferred Reporting Items for Systematic review and Meta-Analysis Protocols) 2015 checklist: recommended items to address in a systematic review protocol*

Section and topic	Item No	Checklist item
**ADMINISTRATIVE INFORMATION**		
Title:		
Identification	1a	Identify the report as a protocol of a systematic review
Update	1b	If the protocol is for an update of a previous systematic review, identify as such
Registration	2	If registered, provide the name of the registry (such as PROSPERO) and registration number
Authors:		
Contact	3a	Provide name, institutional affiliation, e-mail address of all protocol authors; provide physical mailing address of corresponding author
Contributions	3b	Describe contributions of protocol authors and identify the guarantor of the review
Amendments	4	If the protocol represents an amendment of a previously completed or published protocol, identify as such and list changes; otherwise, state plan for documenting important protocol amendments
Support:		
Sources	5a	Indicate sources of financial or other support for the review
Sponsor	5b	Provide name for the review funder and/or sponsor
Role of sponsor or funder	5c	Describe roles of funder(s), sponsor(s), and/or institution(s), if any, in developing the protocol
**INTRODUCTION**		
Rationale	6	Describe the rationale for the review in the context of what is already known
Objectives	7	Provide an explicit statement of the question(s) the review will address with reference to participants, interventions, comparators, and outcomes (PICO)
**METHODS**		
Eligibility criteria	8	Specify the study characteristics (such as PICO, study design, setting, time frame) and report characteristics (such as years considered, language, publication status) to be used as criteria for eligibility for the review
Information sources	9	Describe all intended information sources (such as electronic databases, contact with study authors, trial registers or other grey literature sources) with planned dates of coverage
Search strategy	10	Present draft of search strategy to be used for at least one electronic database, including planned limits, such that it could be repeated
Study records:		
Data management	11a	Describe the mechanism(s) that will be used to manage records and data throughout the review
Selection process	11b	State the process that will be used for selecting studies (such as two independent reviewers) through each phase of the review (that is, screening, eligibility and inclusion in meta-analysis)
Data collection process	11c	Describe planned method of extracting data from reports (such as piloting forms, done independently, in duplicate), any processes for obtaining and confirming data from investigators
Data items	12	List and define all variables for which data will be sought (such as PICO items, funding sources), any pre-planned data assumptions and simplifications
Outcomes and prioritization	13	List and define all outcomes for which data will be sought, including prioritization of main and additional outcomes, with rationale
Risk of bias in individual studies	14	Describe anticipated methods for assessing risk of bias of individual studies, including whether this will be done at the outcome or study level, or both; state how this information will be used in data synthesis
Data synthesis	15a	Describe criteria under which study data will be quantitatively synthesised
	15b	If data are appropriate for quantitative synthesis, describe planned summary measures, methods of handling data and methods of combining data from studies, including any planned exploration of consistency (such as I^2, Kendall's τ)
	15c	Describe any proposed additional analyses (such as sensitivity or subgroup analyses, meta-regression)
	15d	If quantitative synthesis is not appropriate, describe the type of summary planned
Meta-bias(es)	16	Specify any planned assessment of meta-bias(es) (such as publication bias across studies, selective reporting within studies)
Confidence in cumulative evidence	17	Describe how the strength of the body of evidence will be assessed (such as GRADE)

*** It is strongly recommended that this checklist be read in conjunction with the PRISMA-P Explanation and Elaboration (cite when available) for important clarification on the items. Amendments to a review protocol should be tracked and dated.** **The copyright for PRISMA-P (including checklist) is held by the PRISMA-P Group and is distributed under a Creative Commons Attribution Licence 4.0.**

Appendix 2 - Search Strategies

Category	Keywords	MeSH MEDLINE	MeSH Embase	MeSH GlobalHealth
**Natural Hazards**	natural hazard* OR natural disaster* OR extreme adj2 event*	climatic process exp, cyclonic storms exp, droughts exp, floods exp, tidal waves exp, geological phenomena exp, avalanches exp, earthquakes exp, landslides exp, tsunamis exp, volcanic eruptions exp, wildfires exp, natural disasters exp	natural disaster exp, disaster victim exp, earthquake exp, drought exp, flooding exp, hurricane exp, tsunami exp, landslide exp, avalanche exp, wildfire exp, volcano exp	natural disaster exp, hurricanes exp, tornados exp, typhoons exp, droughts exp, floods exp, earthquakes exp, landslides exp, avalanches exp, tsunami exp, volcanos exp, wildfire exp
**Conflicts**	armed conflict* or civil war*	ethnic violence exp, exposure to violence exp, armed conflicts exp, war exposure exp	war exposure exp, ethnic conflict exp	conflict exp, war exp, aggression exp, fighting exp
**Disease**	infectious disease outbreak* OR communicable disease outbreak*	disease outbreaks exp, epidemics exp, communicable diseases exp, diarrhoea, vibrio infections exp, cholera exp, salmonella infections exp, typhoid fever exp, paratyphoid fever exp, leptospirosis exp, Weil disease, measles exp, measles virus exp, meningitis, bacterial exp, meningitis, escherichia coli exp, meningitis, meaningococcal exp, meningitis, pneumococcal exp, respiratory tract infections exp, malaria exp, dengue exp, tetanus exp, clostridium infections exp, haemorrhagic fevers, viral exp, poliomyelitis exp, poliovirus exp, coccidioidomycosis exp, dysentery exp, leishmaniasis, cutaneous exp, leishmaniasis, visceral exp, hepatitis exp, hepatitis a exp, hepatitis e exp	typhoid fever exp, salmonellosis exp, acute hepatitis exp, hepatitis a virus exp, hepatitis e virus exp, hepatitis a exp, hepatitis e exp, leptospirosis exp, measles exp, respiratory tract infection exp, malaria exp, dengue exp, tetanus exp, coccidioidomycosis exp, haemorrhagic fever exp, poliomyelitis exp, dysentery exp, acute diarrhoea exp, meningitis exp, skin leishmaniasis exp, visceral leishmaniasis exp	outbreaks exp, epidemics exp, infectious diseases exp, diarrhoea exp, cholera exp, vibrio cholerae exp, salmonella typhi exp, typhoid exp, salmonella paratyphi exp, paratyphoid exp, hepatitis a exp, hepatovirus a exp, hepatitis e exp, hepatovirus e exp, leptospirosis exp, measles exp, meningitis exp, bacterial meningitis exp, viral meningitis exp, respiratory diseases exp, malaria exp, dengue exp, tetanus exp, coccidioidomycosis exp, leishmaniasis exp, cutaneous leishmaniasis exp, visceral leishmaniasis exp, haemorrhagic fever exp, poliomyelitis exp, dysentery exp

Reporting checklist for protocol of a systematic review.

Based on the PRISMA-P guidelines.

Instructions to authors

Complete this checklist by entering the page numbers from your manuscript where readers will find each of the items listed below.

Your article may not currently address all the items on the checklist. Please modify your text to include the missing information. If you are certain that an item does not apply, please write "n/a" and provide a short explanation.

Upload your completed checklist as an extra file when you submit to a journal.

In your methods section, say that you used the PRISMA-Preporting guidelines, and cite them as:

Moher D, Shamseer L, Clarke M, Ghersi D, Liberati A, Petticrew M, Shekelle P, Stewart LA. Preferred Reporting Items for Systematic Review and Meta-Analysis Protocols (PRISMA-P) 2015 statement. Syst Rev. 2015;4(1):1.

		Reporting Item	Page Number
**Title**			
Identification	#1a	Identify the report as a protocol of a systematic review	1
Update	#1b	If the protocol is for an update of a previous systematic review, identify as such	n/a
**Registration**			
	#2	If registered, provide the name of the registry (such as PROSPERO) and registration number	n/a
**Authors**			
Contact	#3a	Provide name, institutional affiliation, e-mail address of all protocol authors; provide physical mailing address of corresponding author	1

For peer review only

Contribution	#3b	Describe contributions of protocol authors and identify the guarantor of the review	1
**Amendments**			
	#4	If the protocol represents an amendment of a previously completed or published protocol, identify as such and list changes; otherwise, state plan for documenting important protocol amendments	n/a
**Support**			
Sources	#5a	Indicate sources of financial or other support for the review	8
Sponsor	#5b	Provide name for the review funder and / or sponsor	8
Role of sponsor or funder	#5c	Describe roles of funder(s), sponsor(s), and / or institution(s), if any, in developing the protocol	8
**Introduction**			
Rationale	#6	Describe the rationale for the review in the context of what is already known	2
Objectives	#7	Provide an explicit statement of the question(s) the review will address with reference to participants, interventions, comparators, and outcomes (PICO)	3
**Methods**			
Eligibility criteria	#8	Specify the study characteristics (such as PICO, study design, setting, time frame) and report characteristics (such as years considered, language, publication status) to be used as criteria for eligibility for the review	4
Information sources	#9	Describe all intended information sources (such as electronic databases, contact with study authors, trial registers or other grey literature sources) with planned dates of coverage	4
Search strategy	#10	Present draft of search strategy to be used for at least one electronic database, including planned limits, such that it could be repeated	appendix 2

For peer review only

Study records - data management	#11a	Describe the mechanism(s) that will be used to manage records and data throughout the review	4
Study records - selection process	#11b	State the process that will be used for selecting studies (such as two independent reviewers) through each phase of the review (that is, screening, eligibility and inclusion in meta-analysis)	4
Study records - data collection process	#11c	Describe planned method of extracting data from reports (such as piloting forms, done independently, in duplicate), any processes for obtaining and confirming data from investigators	5
Data items	#12	List and define all variables for which data will be sought (such as PICO items, funding sources), any pre-planned data assumptions and simplifications	4
Outcomes and prioritization	#13	List and define all outcomes for which data will be sought, including prioritization of main and additional outcomes, with rationale	5
Risk of bias in individual studies	#14	Describe anticipated methods for assessing risk of bias of individual studies, including whether this will be done at the outcome or study level, or both; state how this information will be used in data synthesis	4
Data synthesis	#15a	Describe criteria under which study data will be quantitatively synthesised	5
Data synthesis	#15b	If data are appropriate for quantitative synthesis, describe planned summary measures, methods of handling data and methods of combining data from studies, including any planned exploration of consistency (such as I2, Kendall's τ)	5
Data synthesis	#15c	Describe any proposed additional analyses (such as sensitivity or subgroup analyses, meta-regression)	5
Data synthesis	#15d	If quantitative synthesis is not appropriate, describe the type of summary planned	5
Meta-bias(es)	#16	Specify any planned assessment of meta-bias(es) (such as publication bias across studies, selective reporting within	5

For peer review only

		studies)	
Confidence in cumulative evidence	#17	Describe how the strength of the body of evidence will be assessed (such as GRADE)	5

The PRISMA-P checklist is distributed under the terms of the Creative Commons Attribution License CC-BY 4.0. This checklist was completed on 20. April 2020 using https://www.goodreports.org/, a tool made by the EQUATOR Network in collaboration with Penelope.ai

For peer review only
